# The Dualistic Effect of COX-2-Mediated Signaling in Obesity and Insulin Resistance

**DOI:** 10.3390/ijms20133115

**Published:** 2019-06-26

**Authors:** Pei-Chi Chan, Min-Tser Liao, Po-Shiuan Hsieh

**Affiliations:** 1 Institute of Physiology, National Defense Medical Center, Taipei 114, Taiwan; 2 Department of Pediatrics, Taoyuan Armed Forces General Hospital, Taoyuan 325, Taiwan; 3 Department of Pediatrics, Tri-Service General Hospital, Taipei 114, Taiwan; 4 Department of Medical Research, Tri-Service General Hospital, Taipei 114, Taiwan

**Keywords:** cyclooxygenase II, prostaglandins, obesity, metabolic syndrome, energy metabolism

## Abstract

Obesity and insulin resistance are two major risk factors for the development of metabolic syndrome, type 2 diabetes and associated cardiovascular diseases (CVDs). Cyclooxygenase (COX), a rate-limiting enzyme responsible for the biosynthesis of prostaglandins (PGs), exists in two isoforms: COX-1, the constitutive form, and COX-2, mainly the inducible form. COX-2 is the key enzyme in eicosanoid metabolism that converts eicosanoids into a number of PGs, including PGD_2_, PGE_2_, PGF_2α_, and prostacyclin (PGI_2_), all of which exert diverse hormone-like effects via autocrine or paracrine mechanisms. The *COX-2* gene and immunoreactive proteins have been documented to be highly expressed and elevated in adipose tissue (AT) under morbid obesity conditions. On the other hand, the environmental stress-induced expression and constitutive over-expression of COX-2 have been reported to play distinctive roles under different pathological and physiological conditions; i.e., over-expression of the *COX-2* gene in white AT (WAT) has been shown to induce de novo brown AT (BAT) recruitment in WAT and then facilitate systemic energy expenditure to protect mice against high-fat diet-induced obesity. Hepatic COX-2 expression was found to protect against diet-induced steatosis, obesity, and insulin resistance. However, COX-2 activation in the epidydimal AT is strongly correlated with the development of AT inflammation, insulin resistance, and fatty liver in high-fat-diet-induced obese rats. This review will provide updated information regarding the role of COX-2-derived signals in the regulation of energy metabolism and the pathogenesis of obesity and MS.

## 1. Introduction

Obesity is not only the most prevalent manifestation but also a therapeutic target for the prevention and treatment of metabolic syndrome and type 2 diabetes mellitus (DM). Previous reports have indicated that cyclooxygenase (COX)-2-derived prostaglandins (PGs) are not only crucially involved in the development of obesity-associated metabolic syndrome but also substantially contribute to the regulation of energy metabolism under different pathophysiological conditions. The production of PGs begins with the liberation of arachidonic acid from membrane phospholipids by phospholipase A2 in response to inflammatory stimuli. Arachidonic acid is converted to PGH2 by the cyclooxygenase enzymes COX-1 and COX-2. COX-2 is mainly an inducible enzyme, which is regulated by growth factors and different cytokines, such as IL-1β, IL-6, or TNFα and is involved primarily in the regulation of inflammation. The *COX-2* gene is located on chromosome 1 and its promoter displays an NFκB response element, as well as other cytokine-dependent, such as IL-6 response elements.

**Figure 1:**
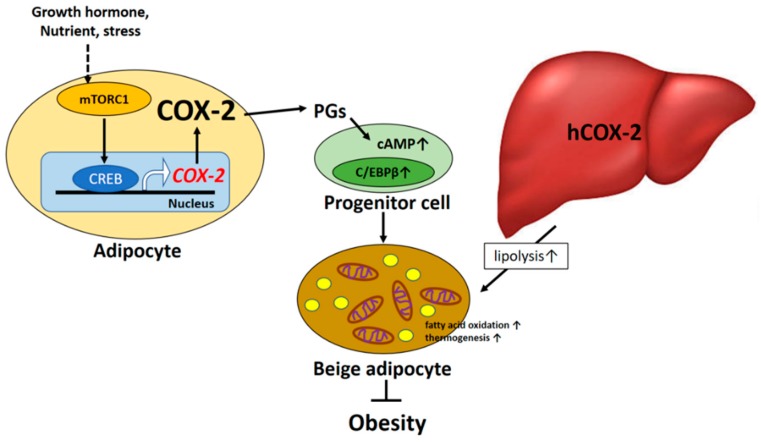
The beneficial effect of COX-2 mediated signaling on obesity and insulin resistance.

**Figure 2:**
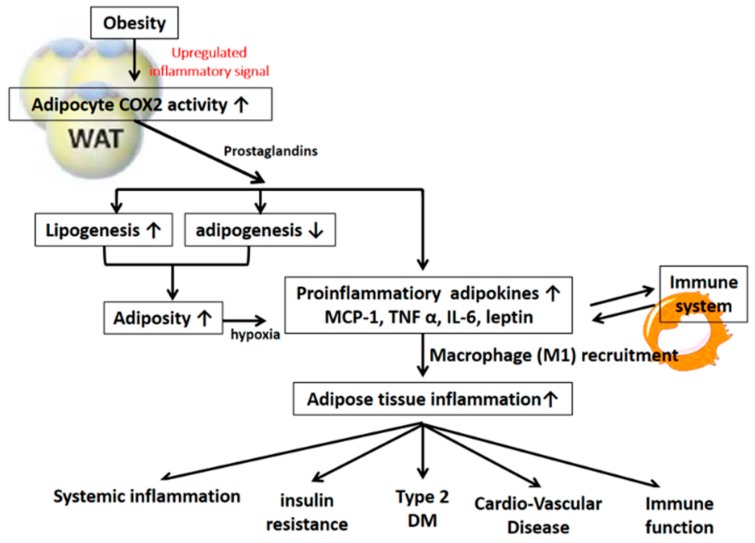
The detrimental effect of COX-2 mediated signaling on obesity and insulin resistance.

There are four principal bioactive PGs that are derived from the COX-2 signaling pathway: PGE_2_, prostacyclin (PGI_2_), PGD_2_, and PGF_2α_. These PGs are ubiquitously produced and act as autocrine and paracrine lipid mediators to maintain local homeostasis in the body. During an inflammatory response, both the level and the profile of PG production changes dramatically. PG levels remain very low in uninflamed tissues but can increase immediately as a result of acute inflammation prior to the recruitment of leukocytes and the infiltration of immune cells. On the other hand, PGs also play the important role in the regulation of vascular tone, cell proliferation, and differentiation and energy metabolism [1,2]. This review describes recent advances in our understanding of the mechanism and function of both COX-2-derived signals in the regulation of energy metabolism as shown in Figure 1 and the pathogenesis of obesity and metabolic syndrome as shown in Figure 2. The final section of this review speculates on future prospects for COX-2-derived PG-based therapies in humans.

## 2. COX-2-Derived PGs and Regulation of Energy Metabolism

### 2.1. Adipose Tissue COX-2-Derived PGs and Regulation of Energy Metabolism

COX-2 is the rate-limiting enzyme in the process of PG synthesis. COX-1- and COX-2-mediated PGs have recently been shown to significantly participate in regulating the recruitment and activation of beige fat cells (the “browning” effect) in mice [3,4]. The over-expression of COX-2 induced de novo browning recruitment in white adipose tissue (WAT) and facilitated systemic energy expenditure, resulting in the retardation of high-fat diet (HFD)-induced obesity in mice [4]. On the other hand, COX-2 activation in WAT has been shown to be a downstream effector of the β-adrenergic receptor signal and is required for the induction of beige biogenesis in WAT depots [3]. The COX-2-derived PG pathway is important in controlling the differentiation of defined mesenchymal progenitors toward the phenotype of brown adipocytes. Notably, the cold-induced expression of UCP1 in inguinal white adipocytes but not in brown adipose tissue (BAT) was repressed in mice with *COX-2* gene deletion in this study, suggesting that the action of COX-2-mediated signaling on adaptive thermogenesis is carried out through a myf-5 independent pathway [3]. However, the possible compensatory mechanism of BAT-mediated adaptive thermogenesis to global *COX-2* gene deletion could not be ignored. Accordingly, several investigations have revealed the molecular mechanism by which local COX-2-derived PG production can affect the browning phenomenon in WAT and systematic energy expenditure. For instance, cold exposure or the β_3_-adrenoreceptor agonist could induce browning through an increase in prostacyclin (PGI_2_) production, which in turn shifts the differentiation of defined mesenchymal progenitors toward a brown adipocyte phenotype [4]. In addition, the micromolar concentrations of carbaprostacyclin (cPGI_2_), a synthetic analog of PGI_2_, have been demonstrated to promote browning of adipocytes in in vitro cell-culture models [5,6]. cPGI_2_-mediated morphological responses facilitated beige/brite differentiation of mouse and human primary progenitor cells from white fat and were causally linked to the priming of thermogenic gene expression [5]. Furthermore, it has been demonstrated that exposure of preadipocytes of WAT origin to PGE_2_ results in a browning effect during the adipocyte differentiation process [7]. PGE_2_ induced the expression of brown markers (UCP1 and PRDM16) in WAT and adipocytes and participates in the differentiation of WAT pre-adipocytes in beige cells [8]. These observations provide supportive evidence that PGs could shift the differentiation of progenitors toward a brown adipocyte phenotype. The findings also suggest that COX-2-mediated PGs act as paracrine signals targeting adipocyte progenitor cells that orchestrate beige adipogenesis. In addition, mTORC1 signaling acts as a key regulator of the COX-2/PG pathway through the phosphorylation of CREB-regulated transcription coactivator 2 (CRTC2) in adipocytes and has been shown to mediate obesity-induced suppression of COX-2 and beige adipogenesis in WAT [9].

In contrast, a recent report demonstrated that cold-induced browning of adipocytes in the mouse inguinal fat pad is not dependent on adipose tissue COX-2 activation. Georgios et al. used mice with global *COX-2* gene deletion, which was achieved postnatally by a tamoxifen-inducible form of Cre-recombinase, bypassing the effect of COX-2 deficiency during development. They also conducted physiological assessments with transgenic mice with global and adipocyte-specific deletions of COX-2, and showed that cold exposure did activate the thermogenesis and browning of inguinal WAT in the absence of increasing COX-2 expression in adipose tissues. Therefore, they concluded that COX-2 lacks a physiological role in both inguinal WAT, gonadal WAT and interscapular BAT of mice undergoing thermogenesis in a cold environment [10]. Overall, although the physiological role of COX-2 activity in adaptive thermogenesis might be compensated for, the regulatory effect of COX-2-derived PGs on adaptive thermogenesis and energy metabolism cannot be ignored.

### 2.2. Hepatic COX-2-Derived PGs and Regulation of Energy Metabolism

Although adult hepatocytes fail to induce COX-2 expression when exposed to pro-inflammatory stimuli, the constitutive expression of the human COX-2 gene in mouse hepatocytes has shown a protective effect against the adiposity, inflammation, and systemic insulin resistance induced by HFD feeding. In addition, human COX-2 transgenic mice exhibited an increase in whole-body energy expenditure due, at least in part, to the induction of thermogenesis and fatty acid oxidation. This study also indicates that COX-2-derived PGs in the liver could be involved in the mechanism underlying the beneficial action against HFD-induced obesity and its associated impaired energy metabolism, inflammation, and insulin resistance by favoring lipid clearance in hepatocytes [11].

## 3. COX-2-Derived PGs and Obesity Associated Complications

Chronic low-grade inflammation and oxidative stress along with obesity and insulin resistance have been speculated to play a central role in the pathogenic mechanism of metabolic syndrome and type 2 diabetes. In Pima Indians, a promotor variant in the inducible *COX-2* gene was previously reported to be linked to the early onset of type 2 DM via its involvement in the inflammatory response and generation of PGs [12]. On the other hand, a causal link between augmented COX-2-dependent vasoconstriction and renal endothelial dysfunction, through the enhanced reactive oxygen species (ROS) generation, has been found in obesity [13]. Moreover, the expression of the *COX-2* gene and the production of ROS were shown to be induced by hyperglycemia in cultured human mesangial cells, which are target cells in diabetic nephropathy. Renal cortical COX-2 protein expression and function were increased in obese Zucker diabetic fatty (ZDF) rats. The changes in expression and activity of COX-2 in ZDF rats were associated with metabolic abnormalities, as well as increased kidney weight and mild proteinuria in the insulin-resistant state. Moreover, selective COX-2 inhibition has been suggested to be potentially capable of preventing or delaying diabetic neuropathy [14]. In clinical studies, Laura et al. demonstrated that, in the breast tissue of obese women, a higher concentration of circulating cytokines promotes greater macrophage COX-2 expression and produces more PGE_2_ [15]. These observations indicate that COX-2-mediated inflammation substantially contributes to relevant complications in subjects with metabolic syndrome and diabetes, further strengthening the importance of COX-2 activation in the development of obesity and obesity-related complications.

### 3.1. COX-2-Derived PGs in the Development of Obesity and Adipogenesis

Obesity induces WAT dysfunction characterized by sustained inflammation and fibrosis, impaired adaptive thermogenesis and increased lipolysis. The *COX-2* gene has been shown to be highly expressed in the subcutaneous adipose tissue of obese humans, and the administration of arachidonic acid and PGE_2_ could stimulate leptin release in the adipose tissue of obese humans [16,17]. Mice that are heterozygous for the *COX-*2** gene develop obesity, thereby suggesting that COX-2 activation is involved in the regulation of body fat metabolism [18]. Obesity is also typically accompanied by increased circulating levels of several cytokines that may further enhance local COX-2 expression. For example, serum concentrations of IL-6 and TNFα are generally increased in obese individuals [19], and these cytokines have been shown to promote PGE_2_ production in multiple cell types via their effects on COX-2 expression [16,20]. Moreover, loss of PG production in adipose tissue by the deletion of adipocyte phospholipase A_2_ (AdPLA) was shown to increase lipolysis and result in a higher energy expenditure with increased fatty acid oxidation within adipocytes. AdPLA knockout mice were shown to be resistant to diet-induced obesity [21].

Recent studies have suggested a link between COX-2 activity and adipocyte differentiation [22,23]. Stable transfection of antisense COX-2 increased 3T3-L1 adipocyte differentiation, accompanied by a decrease in PGE_2_ and PGF_2α_ levels [22]. Moreover, PGE_2_-EP4 signaling has been shown to suppress adipocyte differentiation by affecting PPARγ expression in an autocrine manner [24]. Additionally, PGE_2_ was shown to exert an anti-adipogenic effect via its EP3 receptor [1]. In our recent studies, the diminished gene expression of PPAR-γ and C/EBP-α, markers of adipocyte differentiation, in the epididymal fat of high-fat-induced obese rats, was significantly reversed in those cotreated with a selective COX-2 inhibitor [25]. Nevertheless, another study [17] was conducted with COX-2-deficient mice to examine the role of COX-2-derived PGs as modulators of adiposity. Their data showed that the global knockout of COX-2 could result in a significant reduction in body weight and fat mass. Nevertheless, adipocyte differentiation was impaired in adipose tissue from COX-2-deficient mice, which resulted from the attenuated production of 15d-PGJ_2_ and its precursor PGD_2_ [18]. In addition, the modulation of the activity of COX-2 via a COX-2 selective inhibitor impaired adipocyte differentiation through the inhibition of the clonal expansion phase [23]. Collectively, the results of these investigations indicate that the role of COX-2 activation in adipogenesis remains debated and is needed to be further elucidated.

### 3.2. COX-2-Derived PGs in the Pathogenesis of Adipose Tissue Inflammation and Insulin Resistance

COX-2/PG signaling plays a critical role in regulating adipose tissue inflammation and obesity-induced insulin resistance [16,26,27]. Adipose tissue COX-2 activation (rather than COX-1 activation) was found to crucially contribute to the development of fat-inflammation and obesity-associated insulin resistance, fatty liver, and increased oxidative stress in a HFD-induced rodent model [25,27,28]. Treatment with COX-2 inhibitor significantly suppressed the augmentation of adipose MCP-1 and TNF-α gene expression, macrophage infiltration and fat inflammation in both the visceral and subcutaneous adipose tissue of HFD-induced obese rats [25,27]. COX-2 inhibition also increases the expression of the anti-inflammatory adipokine adiponectin in the WAT of arthritic rats [29]. Similarly, inhibition of COX-2 by selective inhibitors resulted in a reduction in adipose tissue mass development and muscular insulin resistance in murine models of nutritionally-induced obesity [30]. In addition, and in the context of obesity, many immune cells infiltrate or populate adipose tissue and promote chronic low-grade inflammation. COX-2 is expressed in adipocytes along with a variety of immune cells residing in adipose tissue, such as macrophages, neutrophils, and T cells. COX-2-stimulated MCP-1 production plays an important role in immune cell infiltration into adipose tissue and the subsequent inflammatory responses and insulin resistance [16,25,26,27]. The diminishment of macrophage-dependent inflammation has recently been demonstrated in the adipose tissue of COX-2-deficient mice, and this diminishment was combined with a significant reduction in body weight and body fat mass [18]. Furthermore, microsomal PGE synthase-1 (mPGES-1) is an enzyme that specifically catalyzes the final step of PGE_2_ biosynthesis. The mPGES-1 knockout mice fed a HFD had lower body weight gain and reduced adiposity, which were accompanied by a decrease in adipose tissue inflammation compared to the inflammation of littermate control mice [31]. In addition, the upregulation of adipocyte COX-2, PGE_2_, and EP3 signaling, during obesity-associated adipocyte hypertrophy and hypoxia, was causally linked to the augmented gene expression and protein production of inflammatory adipokines. COX-2-PGE_2_-EP3-mediated signaling was also demonstrated to be involved in the production of important chemoattractants, namely, RANTES and MCP-1, as well as in the subsequent induction of macrophage and T-cell chemotaxis [16]. Targeting inhibition of adipocyte COX-2 and EP3 during hypertrophy and hypoxia reversed the release of the augmented proinflammatory adipokines and the diminished adiponectin and suppressed NF-κB and HIF-1α transcription activation in the development of morbid obesity. It has been suggested that adipocyte COX-2 PGE_2_-EP3-mediated signaling is crucially involved in the development of obesity and obesity-associated adipose tissue inflammation and insulin resistance [16]. 

Nevertheless, EP3^−/−^ mice develop a more robust obese phenotype and insulin resistance when fed a HFD, suggesting that the EP3 receptor-mediated signal might also be involved in the development of adiposity [32]. On the other hand, IL-1β stimulates expression of PG-synthesizing enzymes COX-2 and PGF2α and PGE2 release as well as adipose tissue inflammation and impaired adipocyte differentiation in human preadipocytes, which did not affect after treatment with COX-2 inhibitor, NS-398. It is suggested that IL-1β is one of the upstream regulator of COX-2-mediated signaling [33]. 

In addition, several studies conducted with humans and rodents have supported the theory that macrophage polarization crucially contributes to the pathogenesis of obesity complications [34]. M1-like macrophages are closely linked to the development of inflammation, obesity, and insulin resistance, while M2-like macrophages are associated with a reduction in both obesity and insulin resistance [35]. We recently demonstrated that the adipose COX-2- macrophage migration inhibitory factor (MIF)-mediated inflammatory cycle between hypertrophic and hypoxic adipocytes, and macrophages, crucially contributes to the proinflammatory phenotypic switch of macrophages, via CD74, during the development of obesity-associated adipose tissue inflammation. MIF has been found to be an important endogenous mediator of COX-2 expression in a number of different cell types, and MIF is also affected by COX-2 activity, which is necessary for several of its pro-inflammatory and pro-tumorigenic activities [36,37]. Our study provides supportive evidence that adipocyte COX-2 activation upregulates MIF production via NFκB activation, during the development of adipocyte hypertrophy and hypoxia. Then, at an early stage of adipose tissue inflammation, during the development of obesity, adipocyte MIF secretion directly facilitates M1 macrophage polarization via CD74, which is reported to be part of the MIF receptor complex [38]. The accumulation of macrophage-derived MIF in the advanced stages of morbid obesity could further exacerbate both adipocyte inflammation and macrophage polarization. This vicious cycle would result in the exaggeration of adipose tissue inflammation and associated insulin resistance in morbid obesity. These reports suggest that COX-2-mediated signaling in adipose tissue predominantly contributes to the initiation and maintenance of adipose tissue inflammation and insulin resistance in the state of obesity.

### 3.3. Hepatic COX-2-Derived PGs and Obesity and Insulin Resistance

COX-2 activation has been reported to be involved in the pathogenesis of different hepatic diseases, ranging from non-alcoholic fatty liver disease (NAFLD) to hepatocellular carcinoma. NAFLD is recognized as the hepatic manifestation of metabolic syndrome and is strongly associated with hyperlipidemia, diabetes mellitus, obesity, and insulin resistance. The role of COX-2-derived PGs in NAFLD remains controversial. Some reports showed that PGs may facilitate lipid accumulation in hepatocytes, and others provided opposite evidence that PGE_2_ suppresses de novo lipogenesis [25,27,39]. Our previous studies showed that cotreating rats fed a high-fructose diet or a HFD with COX-2 inhibitors could significantly improve systemic insulin resistance and fatty liver [25,27,40]. These findings are in agreement with those of with another report using murine models of non-alcoholic steatohepatitis (NASH), induced by a HFD- or methionine- and choline-deficient (MCD) diet, by which it was shown that the increases in hepatic COX-2 expression, and the associated inflammation in the NASH model, could be attenuated by celecoxib treatment [41]. The study conducted with hepatocyte-specific COX-2 transgenic mice (hCOX-2-Tg) showed that hCOX-2-Tg mice have lower levels of NASH hallmarks under MCD diet feeding. The researchers suggested that sustained hepatic PGE_2_ production plays a protective role against the development of NASH and hepatic fibrosis [42]. In addition, miR-183 in liver cells has been shown to be repressed after COX-2 expression and/or in COX-2-Tg hepatocytes; this repression is important for the preservation and even potentiation of the insulin signaling pathway after various hepatic challenges [43]. These observations suggest that hepatic COX-2-derived PGs might play a protective role against fatty liver and liver inflammation. It has also been suggested that the enhancement of COX-2-derived PG signaling under physiological conditions could protect against the development of NASH. Conversely, COX-2 activation could trigger the opposite deteriorating effect on the progression of NASH during the onset of metabolic syndrome and type 2 diabetes.

### 3.4. COX-2-Derived PGs in Obesity Associated Cardiovascular Diseases

Metabolic syndrome is associated with an increased risk for the development of cardiovascular disease. COX-2-derived PGs are involved in the pathophysiology of vascular diseases, such as atherosclerosis and hypertension [44]. Another study investigated the presence of prostanoid metabolic enzymes in atherosclerosis. The study revealed that the expression of COX-1 was exhibited both in normal arteries, and in atherosclerotic lesions, while COX-2 was observed in only atherosclerotic plaques. In these lesions, COX-2 is mainly expressed in macrophages located in the plaque shoulder and consequently activates chemotaxis, the production of inflammatory cytokines and the modification of vascular permeability [45]. The mRNA expression of COX-2 in plaques of individuals with type 2 DM was found to contribute to the inflammatory process associated with atherosclerotic plaque formation [46]. Studies in mice have shown an increase in COX-2 expression in mice with atherosclerosis and demonstrated that celecoxib, a selective COX-2 inhibitor, can prevent the evolution of atheroma lesions [45]. Nevertheless, inhibition of COX-2 activity in patients with atherosclerosis has also been reported to be detrimental with respect to plaque stability and to be associated with increased cardiovascular events and mortality [47]. This controversy could be explained by the fact that COX-2 inhibitors not only inhibit the inflammatory action of PGs but also inhibit the antithrombotic effects of PGI_2_ and thus increase the risk of cardiovascular complications.

## 4. Clinical Implication

To date, COX-2 is the target of a class of nonsteroidal anti-inflammatory drugs (NSAIDs) that includes celecoxib from Pfizer Inc. Another COX-2 inhibitor, rofecoxib from Merck & Co. Inc. was withdrawn from the market in 2004 because of concerns over increased risk of cardiovascular events associated with long-term use. In clinical studies, González-Ortiz et al. reported that specific inhibition of COX-2 by celecoxib increased insulin sensitivity in overweight or obese subjects who were treated with celecoxib for 4 weeks [48]. In addition, the use of combination therapy could be a promising new area in obesity treatment, similar to diabetes, hypertension [49] and osteoarthritis combination therapies [50]. It was demonstrated that combined therapy with metformin and a COX-2 inhibitor synergistically and effectively improved adipose tissue inflammation and systemic metabolic abnormalities in obese rats [51]. Recent evidence has shown the clinical benefit of an aromatase inhibitor/COX-2 inhibitor combination treatment for obese, postmenopausal breast cancer patients [49,52]. Nevertheless, PGE_2_ and its analogs are clinically used to ameliorate mild hypercholesterolemia among other liver pathologies [53]. Collectively, targeting COX-2-derived PGs, for the prevention and treatment of obesity and metabolic syndrome, is of clinical importance. However, different strategies should be used for different clinical conditions.

## 5. Conclusions and Future Direction

Obesity is a serious health problem of the 21st century and one of the major causes of metabolic syndrome. Obesity represents a major epidemic as its prevalence is increasing both in developed and developing countries. However, an effective therapeutic strategy for obesity-associated cardiometabolic abnormalities is not yet available. COX-2-derived PGs have been shown to increase the levels of lipid-burning BAT and could thus trigger weight loss to prevent the development of obesity. However, it is difficult to ignore the fact that COX-2 activation has been shown to be one of the key factors contributing to the inflammation associated with obesity. The overview of animal and in vitro studies of COX-2 mediated signaling in energy metabolism and the development of obesity and insulin resistance are summarized in Table 1 and Table 2. The challenge will be to develop COX-2-targeting compounds that boost the energy-consuming pathway in adipose tissue without increasing COX-2-related inflammation. In addition, COX-2-derived PGs could be potential therapeutic targets for the treatment of obesity-induced insulin resistance and its related disorders. Nevertheless, the cellular and molecular mechanisms and the potential interplay of four principle COX-2 derived PGs such as PGE_2_, prostacyclin (PGI_2_), PGD_2_, and PGF_2α_ and their receptor-mediated signaling in regulation of energy metabolism and are still unclear, and needs to be further elucidated (Table 3). 

## Figures and Tables

**Table 1:** Overview of animal studies targeting COX-2 -mediated signaling pathway.

	Target	Gene	Model	Effects	Reference
Energy metabolism	global	*Cox-2*	COX2 KO mice	Cold-induced expression of UCP1 in inguinal white adipocytes was repressed	[3]
			(B6;129P2 Ptgs2 tm1Unc)		
	global	*Cox-2*	COX2 KO mice	BAT characteristics were diminishedin WAT of CL-treated COX-2–/– mice	[4]
			(B6;129P2 Ptgs2 tm1Unc)		
	global	*Cox-2*	K5COX2 mice (line 675+/+)	induced de novo BAT recruitment in WAT, increased systemic energy expenditure, and protected mice against HFD–induced obesity.	[4]
			(overexpressing the COX-2 gene under the control of the promoter for the keratin 5 gene)		
	global	*Cox-2*	COX2 KO mice	Deletion of COX-2 fails to suppress cold-induced browning and UCP-1 expression in AT	[10]
			(COX-2 flox/flox mice; Cre-ER,tamoxifen-inducible form of Cre-recombinase)		
	adipose tissue	*Cox-2*	adipocyte-specific COX-2 KO mice	no alteration in metabolite excretion under basal conditions and augment their formation in response to cold	[10]
			(COX-2 flox/flox mice; adiponectin-Cre)		
Obesity and insulin resistance	global	*AdPLA*	AdPLA-null mice	increases lipolysis and prevents obesity induced by HFD feeding	[21]
			(C57BL/ 6J)		
	global	*Cox-2*	COX-2−/− mice	Macrophage-dependent AT inflammation was reduced	[18]
			(C57BL/6J × 129/Ola (C57/129))		
	global	*mPGES-1*	mPGES-1−/− (KO) mice	reduces diet-induced low-grade inflammation and adiposity	[31]
			(DBA/11ac J)		
	global	*EP3*	EP3−/− mice	increased epididymal fat mass and adipocyte size and macrophage infiltration	[32]
			(C57BL/6J)		
	liver	*Cox-2*	hepatocyte-specific COX-2 transgenic mice	lower grades of steatosis, inflammation and reduced recruitment and infiltration of hepatic macrophages	[43]
			(B6D2/OlaHsd)		
	global	*Cox-2/PGE2/EP3*	HFD induced obese mice	increased obesity-associated AT inflammation and systemic insulin resistance	[16,25,26]

**Table 2:** Overview of in vitro studies targeting COX-2-mediated signaling pathway.

	Target Gene or Protein	Method	Model	Effects	Reference
Energy metabolism	COX	COX inhibitor, indomethacin	Rb–/– MEFs	COX activity is required for induction of UCP-1	[3]
			(embryo fibroblasts (MEFs) lacking the retinoblastioma (Rb) gene)		
	cPGI2	norepinephrine (NE) treatment	Differentiation of primary human mesenchymal cells	NE-induced cPGI2 shifts the differentiation of WAT mesenchymal progenitors toward a brown adipocyte phenotype	[4,5]
	cPGI2	norepinephrine (NE) treatment	beige/brite progenitor cells	cPGI2 induces a broad thermogenic gene expression program in adipocyte progenitors	[5]
			(Lin−CD29+CD34+Sca-1+ cells)		
	cPGI2	cPGI2 treatment	hMADS	activates white to brite adipocyte conversion	[6]
			(human multipotent adipose-derived stem cells)		
	mPGES-1	mPGES-1 siRNA	3T3-L1 adipocytes	mPGES-1 as a key regulator of white-to-brown adipogenesis	[7]
	PGE2	PGE2 treatment	adipocytes isolated from human omental WAT	PGE2 increased the expression of UCP1 and PRDM16 in adipocytes	[8]
Obesity and insulin resistance	*Cox-2*	COX-2 shRNA and COX-2 inhibitor, NS398	3T3-L1 adipocytes	The suppressive effect of COX-2 inhibition was noted in the release of pro-inflammatory adipokines into the medium from the hypertrophy adipocytes	[16]
	*Cox-2*	lentivirus derived shCOX-2 or COX-2 cDNA	SGBS adipocytes	adipocyte COX-2 activation up-regulates MIF production during thedevelopment of hypertrophy and hypoxia	[38]
	*Cox-2*	COX-2 inhibitor,sc-58236	3T3-L1 adipocytes	inhibition of the COX-2 enzyme impairs adipocyte differentiation	[23]
	*Cox-2*	COX-2 inhibitor, NS398	mouse embryonic fibroblasts (MEF)	COX-2-derived PGE2 suppresses adipocyte differentiation in MEF cells	[24]
	EP3		mouse embryonic fibroblasts (MEF) isolated EP3–/– mice WAT	activation of EP3 receptor suppressed adipogenesis and lipolysis	[1]

**Table 3:** Opportunities for future research.

1	The cellular and molecular mechanisms and the potential interplay of COX-2 derived PGs in control of energy metabolism and the development of obesity and insulin resistance.
**2**	The detailed mechanisms regarding the role of PGs and their receptors in the development of these COX-2 mediated phenomenon.
**3**	The therapeutive strategy to develop COX-2 targeting compounds which could boost energy expenditure without trigger COX-2-mediated inflammation
**4**	To dissect the role of COX-2 dependent and independent adaptive thermogenesis and their impact on energy homeostasis
**5**	Clinical application of selective COX-2 activator in prevention and treatment of NAFLD
